# Saffron supplements modulate serum pro-oxidant-antioxidant balance in patients with metabolic syndrome: A randomized, placebo-controlled clinical trial

**Published:** 2015

**Authors:** Tayyebeh Kermani, Seyyed Hadi Mousavi, Maryam Shemshian, Abdolreza Norouzy, Mohsen Mazidi, Atefeh Moezzi, Toktam Moghiman, Majid Ghayour-Mobarhan, Gordon A. Ferns

**Affiliations:** 1 *Department of Anatomy and Cell biology, Birjand University of Medical Sciences, Birjand, Iran*; 2 *Pharmacological Research Center of Medicinal Plant, Faculty of Medicine, Mashhad University of Medical Science, Mashhad, Iran*; 3 *Biochemistry of Nutrition Research Center, School of Medicine, Mashhad University of Medical Science, Mashhad, Iran*; 4 *Institute of Genetics and Developmental Biology, University of Chinese Academy of Sciences, Beijing, China*; 5 *Cardiovascular Research Center, Faculty of Medicine, Mashhad University of Medical Science, Mashhad, Iran*; 6 *Division of Medical Education, Brighton & Sussex Medical School, Rm 342, Mayfield House, University of Brighton, BN1 9PH, United Kingdom *

**Keywords:** *Metabolic syndrome*, *prooxidant-antioxidant balance*, *Saffron*

## Abstract

**Objectives:**

We have investigated the effect of a saffron supplement, given at a dose of 100 mg/kg, on prooxidant-antioxidant balance (PAB) in individuals with metabolic syndrome.

**Materials and Methods:**

A randomized, placebo-controlled trial design was used in 75 subjects with metabolic syndrome who were randomly allocated to one of two study groups: (1) the case group received 100mg/kg saffron and (2) the placebo control group received placebo for 12 weeks. The serum PAB assay was applied to all subjects before (week 0) and after (weeks 6 and 12) the intervention.

**Results:**

There was a significant (p=0.035) reduction in serum PAB between week 0 to week 6 and also from week 0 to week 12.

**Conclusion:**

Saffron supplements can modulate serum PAB in subjects with metabolic syndrome, implying an improvement in some aspects of oxidative stress or antioxidant protection.

## Introduction

Metabolic syndrome is a clustering of hypertension, glucose intolerance, elevated triglycerides and low level of high density lipoprotein (HDL) cholesterol (Eckel et al., 2005). The IDF (International Diabetes Federation) has defined four criteria for identification of metabolic syndrome: (1) a raised trigriceride (TG) level, ≥ 150 mg/dL (1.7 mmol/L), (2) a reduced HDL cholesterol level, < 40 mg/dL (1.03 mmol/L) in males and < 50 mg/dL (1.29 mmol/L) in females, (3) a raised blood pressure, systolic BP≥ 130 or diastolic BP≥ 85 mm Hg and (4) a raised fasting blood glucose (FBG), ≥ 100 mg/dL (5.6 mmol/L) (Alberti et al., 2006 ▶ ).

Oxidative stress plays a critical role in the initiation and progression of atherosclerosis (Alamdari et al., 2008 ▶). The imbalance between oxidative stress and the anti-oxidant defense may be due to elements that lead to an increased formation of reactive oxygen species (ROS), and this augment is considered as the key factor in the development of cardiovascular diseases (CVD) (Rahsepar et al., 2012 ▶). 

*Crocus Sativus*, commonly known as saffron is a perennial stemless herb of the Iridaceae family, wildly considered as a potential therapeutic agent in of the treatment of various diseases (Nair et al., 1991 ▶). Saffron and its active constituents have also been proven to have properties that improve neural function and promote oxygen delivery to the tissues (Hosseinzadeh et al., 2009 ▶). Saffron has also been reported to have antioxidative and hypolipidemic effects (Gong et al., 2001 ▶)

Oxidative stress is an imbalance between the production of pro-oxidants and antioxidant defense in favor of prooxidants. It is typically associated to augmented formation of ROS, and is thought to play a pivotal role in the pathogenesis and development of CVD and its related complications. It is suggested that markers of oxidative stress may also be predictors of cardiovascular events (Kaminski et al., 2002 ▶ ; Sadeghnia et al., 2013 ▶).

ROS and neutrophils are key players in reperfusion injury, interacting at multiple sites with each other as along with other elements such as the complement system, endothelial cells, macrophages, lymphocytes and myocytes (Kaminski et al., 2002 ▶). Moreover, data have shown a role for ROS in neointimal thickening (Lafont et al., 1995 ▶) and vascular remodeling (Nunes et al., 1995 ▶) late after arterial injury (Tardif et al., 1997 ▶). It has also been reported that isoprostanes, which are a measure of lipid peroxidation, are associated with increased risk of CVD and a number of cardiovascular risk factors (Vassalle et al., 2004 ▶). In patients with CVD, raised levels of oxidative stress status [superoxide anion (O2^•−^) and malonaldehyde (MDA)] and reduced protective superoxide dismutase (SOD) activities have both been reported (Kotur-Stevuljevic et al., 2007 ▶).

Because of the importance of pro-oxidant–antioxidant balance in the development and pathogenesis of CVD, we have investigated the changes in serum pro-oxidant–antioxidant balance (PAB) after saffron consumption in subjects with metabolic syndrome.

## Material and Methods

We undertook a randomized and double-blind clinical trial design over a period of 12 weeks. The investigation was conducted in the Nutrition Clinic, Ghaem Hospital, Mashhad, Iran, between September 2010 and March 2011.

**Participants**

A total of 75 subjects with metabolic syndrome (as defined by International Diabetic Federation criteria 2005), who were between 18-75 years old, were recruited. Patients with systemic diseases (such as AIDS and rheumatoid arthritis), or who were pregnant or breast feeding were excluded. Participants were provided with written and verbal information about the study. All patients provided written informed consent and the protocol satisfied Mashhad University of Medical Sciences Ethics Committee requirements. 

**Study design**

Patients were randomly divided into 2 groups using a computer-generated code: (1) the case group received a capsule of saffron 100 mg/kg/day (50 mg twice a day) (n=35); the placebo control group received a capsule of placebo (twice a day) (n=35), for 12 weeks. Out of 70 subjects originally allocated to the two groups, fifty six subjects completed the trial and fourteen participants were withdrawn (one due to symptoms of possible saffron induced allergy, two due to new pregnancies, two due to undergoing routine surgery and nine did not participate).

All patients were given dietary advice based on American Heart Association (AHA) guidelines during the study. Compliance was monitored every three weeks by visiting each subject and counting capsules. Subjects who did not take their capsules or who were intolerant were excluded from the study. 

**Saffron capsule preparation**

*Crocus sativus* L. stigma was supplied by the Novin Saffron Co. (Mashhad, Iran). It was formulated as a capsule containing 50 mg of dried saffron stigma. Placebo capsules were matched in size, shape and volume of content and manufactured by the same company.

**Demographic and anthropometric data**

For all patients, anthropometric parameters including weight, height, waist circumference (WC) and hip circumference were determined using a standard protocol after an overnight fasting. Height was measured without shoes to the nearest 0.1 cm. Weight was measured in light clothing without shoes to the nearest 0.1 kg. Hip circumference was measured at the level of maximum extension of the buttocks and waist circumference was measured mid-way between the lateral lower rib margin and the iliac crest with the scale to the nearest ±0.1 cm. Moreover, blood pressure was measured while the patients were seated and rested for 15 min, using a standard protocol.

**Blood sampling**

Blood samples were collected from each subject in the morning after a 12 h fasting. Haemolysed samples were excluded from analysis. After separation, aliquots of serum were frozen at −80 °C and kept until analysis. 

**Laboratory Assays**

A full fasting lipid profile comprising of total cholesterol (Bio system S.A, Spain), triglycerides (Bio system S.A. , Spain), high-density lipoprotein cholesterol (HDL-C) (Pars Azmun company, Iran) and low-density lipoprotein-cholesterol (LDL-C) (Pars Azmun company, Iran) was determined for each subject. Serum lipid and fasting blood glucose (FBG) concentrations were measured enzymatically using commercial kits (Pars Azmun Company, Iran). Dyslipidaemia was defined as TC≥ 200mg/dl (5.18 mmol/l), LDL-C≥ 130 mg/dl (3.36 mmol/l), or TG≥ 150 mg/dl (1.69 mmol/l), or HDL-C< 40 mg/dl (1.03 mmol/l) in men and < 50 mg/dl (1.30 mmol/l) in women according to the Third Report of the National Cholesterol Education Program.

**Serum pro-oxidant–antioxidant balance (PAB) assay**

A modified PAB assay was applied based on a previously described method (Falsoleiman et al., 2011 ▶). The standard solutions were prepared by mixing varying proportions (0–100%) of 250 µl hydrogen peroxide with 3 mM uric acid (in 10 mM NaOH). TMB powder (60 mg) was dissolved in 10 mL DMSO. For preparation of TMB cation solution, 400 µl of the TMB/DMSO solution was added to 20 mL of acetate buffer (0.05 M buffer, pH 4.5); Then, 70 µl of fresh chloramine T (100 mM) solution was added to the mixture. The solution was mixed well and incubated for 2 h at room temperature in dark. Next, 25 U of peroxidase enzyme solution was added to 20 mL of TMB cation solution, nd stored at -20^o^C. In order to prepare TMB solution, 200 µl of TMB/DMSO was added to 10 mL of acetate buffer (0.05 M buffer, pH 5.8) and the working solution was prepared by mixing 1 mL TMB cation solution with 10 mL of TMB solution. This working solution was incubated for 2 min at room temperature in dark and used immediately. Ten microliters of each sample, standard or blank (distilled water) were mixed with 200 µL of working solution in each well of a 96-well plate, which was then incubated in dark at 37^o^C for 12 min. At the end of the incubation time, 100 µL of 2 N HCl was added to each well, and the optical density (OD) was measured at 450 nm using an ELISA reader with a reference wavelength of 620 or 570 nm. A standard curve was plotted from the values relative to the standard samples. The values of the PAB are expressed in arbitrary units, asthe percentage of hydrogen peroxide in the standard solution. The values of the unknown samples were then calculated based on the values obtained from the above-mentioned standard curve.

**Statistical analysis**

All statistical analyses were performed using SPSS for Windows™, version 11.5 software package (SPSS Inc., Chicago, IL, USA). Data were assessed for normality using the Kolmogorov-Smirnov test. Data were expressed as mean±SD or median and interquartile range. Group comparisons were made using ANOVA or Kruskal–Wallis test. Data that were normally distributed were analyzed using one-way analysis of variance (ANOVA). Data that were found not to be normally distributed were analyzed using non-parametric Kruskal–Wallis test. Categorical data were compared using Chi-square test. A two-sided p value< 0.05 was considered as statistically significant. 

## Results

**The baseline characteristics in case and control groups**

No significant differences in baseline characteristics were identified between patients randomly assigned to the case or the control group regarding (*p*> 0.05) (Table 1). 

**Table 1:** Baseline characteristics

	**Case** **Group ** **(n = 26)**	**Placebo control group** **(n = 30)**
**Women (n)**	21	19
**Men (n)**	5	11
**Age (year)**	42.19 ± 11.52	43.60 ± 9.05
**Weight (kg)**	80.63 ± 11.65	79.30 ± 16.43
**Height (cm)**	161.65 ± 9.53	161.90 ± 7.30
**WC (cm)**	105.76 ± 9.01	103.36 ± 12.09
**HC (cm)**	115.61 ± 9.49	112.60 ± 10.89
**Smokers % (n)**	11.5(2)	10(2)
**Diabetics % (n)**	23(5)	20(4)
**Hypertensive % (n)**	23(5)	23.3(5)
**Dyslipide** **mic % (n)**	23(5)	23.3(5)
**Triglycerides (mg/dl)**	139.76 ±70.14	139.00 ± 73.52
**Total cholesterol (mg/dl)**	214.15 ±27.30	227.16 ± 33.34
**HDL-C (** **mg/dl)**	39.03 ± 5.34	39.13 ± 8.00
**LDL-C (mg/dl)**	120.03 ± 30.01	125.16 ± 22.33
**FBS (mg/dl)**	109.69 ± 24.02	108.33 ± 22.26
**SBP (mmHg)**	117.3 ± 1.04	116.1 ± 0.94
**DBP (mmHg)**	76.9 ± 1.04	77.8 ± 1.09

WC, waist circumferences; HC, hip circumferences; HDL-C, high-density lipoprotein cholesterol; LDL-C, low-density lipoprotein cholesterol; FBS, fasting blood sugar; DBP; diastolic blood pressure, SBP; systolic blood pressure. Values are expressed as mean±SD, or median and interquartile range. Chi-square, one-way analysis of variance (ANOVA), and Kruskal–Wallis tests were used to compare qualitative and quantitative (normal and non-normal) variables, respectively.

**Effect of saffron on PAB**

A one-way repeated measures analysis of variance showed a significant effect of the case (saffron) group protocol on PAB values (p=0.029). In the saffron group, post-hoc comparisons showed significant changes of PAB values between week 0 and 6 and also week 0 and 12. The difference between the two groups was significant at the endpoint (week 12) (p=0.035) (Table 2).

## Discussion

CVD is now the most prevalent cause of death globally. Metabolic syndrome that refers to a clustering of cardiovascular risk factors has been generally acknowledged as a simple clinical tool for earlier recognition of an increased risk of atherosclerotic cardiovascular disease (Grundy et al., 2008 ▶; Nesto, 2003 ▶).

**Table 2:** Effect of saffron on serum pro-oxidant balance (measured in HK units).

	**Week 0**	**Week 6**	**Week 12**
**Case group (n=26)**	94.03±28.97	86.87±34.45*	80.67±23.67*
**Control group (n=30)**	91.03 ±32.34	90.52 ±27.23	86.84±22.50

* a one-way repeated measures analysis of variance showed a significant effect of the case (saffron) group protocol on PAB values ( p=0.029).

To our knowledge this relatively small preliminary randomized placebo-controlled study is the first investigation on the effect of saffron on PAB values in humans. Our results indicated a significant effect for 100 mg/kg saffron on PAB values in patients with metabolic syndrome as it was observed that after 12 weeks of treatment, PAB values improved.

Studies in animal models have revealed the potential of saffron constituents in the management of atherosclerosis (He et al., 2007 ▶; He et al., 2005 ▶). These results suggest that saffron may have potentially useful antioxidant properties in interventional studies in human. However, further studies will be required to examine the human application of saffron against oxidative stress-induced CVD.

Crocetin, a natural carotenoid compound found in the stigmas of saffron (*C. sativus* L.) like other carotenoids has the potential of being an effective treatment for diseases related to ROS, such as stroke, ischemia-reperfusion injury, and memory impairment. Radical scavenging effects as well as learning and memory-improving properties (Abe et al., 1999 ▶; Zhang et al., 1994 ▶) and elevation of oxygen diffusion in different tissues (Rı´os et al., 1996 ▶) have also been described. Furthermore, the carotenoid components of saffron, recognized as biological antioxidants, play vital roles in human health through protection of cells and tissues from damaging effects of free radicals and singlet oxygen (Edge et al., 1997 ▶; Palozza et al., 1992 ▶). Moreover, in recent years, it has been suggested that crocetin might be an effective antioxidant against oxidative stress in a hemi-parkinsonian rat model (Ahmad et al., 2005 ▶).

Crocin, another major chemical component of saffron, protects cells from oxidative stress by scavenging free radicals such as superoxides (Bors et al., 1982 ▶). Nair et al. reported that saffron augmented intracellular levels of condensed glutathione and suggested that saffron had antioxidant activity (Nair et al., 1992 ▶). 

Yoshino et al. showed that crocetin reduced oxidative stress in isolated brain cells by acting as a scavenger of reactive oxygen species (Yoshino et al., 2011 ▶). They found that the crocetin significantly reduced oxidative stress especially through scavenging hydroxyl radical (*in vitro* and *ex vivo*). Papandreou et al. investigated the antioxidant property of saffron in saffron-treated mice (Papandreou et al., 2011 ▶) considering the effects of a daily intraperitoneal administration of saffron for seven days, on cognitive functions in both healthy adult (4 months old) and aged (20 months old) male Balb-c mice (n=8/group) using the passive avoidance test. They found that saffron and crocetin provided strong protection in maintaining cell viability, repressing ROS production and decreasing caspase-3 activation. They concluded that, crocetin is a unique and potent antioxidant, capable of mediating the *in vivo* effects of saffron (Papandreou et al., 2011 ▶).

Limitations of the present study were the small number of participants and the short follow up period that should be considered in future studies. 

The results of this study confirm the efficacy of saffron (*C. sativus* L.) in modulating the value of serum PAB in patients with metabolic syndrome. It is therefore possible that saffron may be of value in preventing some of the ROS-related cellular processes associated with atherogenesis. Further studies should be undertaken in order to determine the exact constituent of saffron that has the greatest antioxidant effect, and the dosage required for optimum effect on PAB. A large-scale trial is yet warranted, perhaps followed by some clinical end-point studies.
